# Reply to: Commentary on “The Impact of Diabetes and Metabolic Syndrome Burden on Pain, Neuropathy Severity and Fiber Type”

**DOI:** 10.1002/acn3.70150

**Published:** 2025-10-07

**Authors:** Long Davalos, Brian C. Callaghan, Amro M. Stino

**Affiliations:** ^1^ Department of Neurology Kansas University Medical Center Kansas Missouri USA; ^2^ Department of Neurology University of Michigan School of Medicine Ann Arbor Michigan USA

**Keywords:** cryptogenic sensory polyneuropathy, diabetic neuropathy, idiopathic neuropathy, metabolic syndrome, metabolic syndrome burden

We thank Dr. Khalilizad and colleagues for their thoughtful comments on our article [1, 2] and the opportunity to address the concerns raised. Many of these points were acknowledged as limitations in our original manuscript, but we appreciate the chance to elaborate further.

## 1 Selection and Missing Data Bias

We agree that recruitment from tertiary centers limits external validity and may overrepresent individuals with more severe health conditions. This may explain the high prevalence of metabolic syndrome (MetS) observed in our cohort (62.8%). We also acknowledge that the 27% missing lipid data could introduce selection bias, which may either inflate or attenuate the associations observed.

## 2 Cross‐Sectional Design and Reverse Causality

We concur that the cross‐sectional design precludes definitive conclusions regarding temporality and causality. The lack of data on lifestyle changes and neuropathic pain medication use represents a source of residual confounding that could influence the observed pain outcomes.

## 3 Exposure Misclassification

We agree that central obesity, best measured by waist circumference, DEXA, or MRI of the abdomen, would be a more accurate indicator of visceral adiposity than BMI ≥ 27 kg/m^2^. Unfortunately, none of these measures were available in the registry. Nevertheless, BMI ≥ 27 kg/m^2^ has been validated as a surrogate marker for metabolic risk [3]. Additionally, although hypertension and glycemic status were based on single measurements—an acknowledged limitation—these were cross‐verified with chart review to confirm existing diagnoses and reduce misclassification.

## 4 Outcome Misclassification

We acknowledge that defining pain as a binary variable (yes/no) using any Numeric Rating Scale (NRS) score > 0 may conflate mild discomfort with clinically meaningful neuropathic pain. To address this, we also analyzed pain as a continuous variable, allowing more nuanced comparison between groups. Regarding fiber‐type classification, we adopted a clinical approach consistent with ACTTION criteria for idiopathic distal symmetric polyneuropathy [4]. While more objective assessments such as nerve conduction studies (NCS) and intraepidermal nerve fiber density (IENFD) were only available in a subset of participants, sensitivity analyses in this subgroup yielded similar results, lending support to our findings.

## 5 Residual Confounding

We agree that not adjusting for neuropathic pain medications is a limitation, as these could influence both pain prevalence and intensity. However, diabetes duration and HbA1c were not included because MetS burden (scored 0–4) was analyzed independently of diabetes status. Although waist circumference was not available, it correlates with BMI [5], and BMI was included in our analysis. Regarding alcohol intake, patients for whom alcohol was considered a likely cause of neuropathy were excluded, thereby minimizing its potential as a confounder.

## Author Contributions

Long Davalos: conceptualization, methodology, writing – original draft, visualization. Brian C. Callaghan: conceptualization, methodology, writing – review and editing, visualization, supervision. Amro M. Stino: conceptualization, methodology, writing – review and editing, visualization, supervision.

## Conflicts of Interest

B.C.C. consults for DynaMed, performs medical legal consultations including consultations for the Vaccine Injury Compensation Program, and receives research and editorial support from the American Academy of Neurology. A.M.S. has consulted for Argenx, CSL Behring, Takeda, Sanofi, Immunovant, Annexon, and has received research support from Bristol Myers Squibb and the GBS‐CIDP Foundation. L.D. has no relevant conflicts of interest to disclose.

## Linked Articles

This article is linked to https://onlinelibrary.wiley.com/doi/10.1002/acn3.70151.

## Data Availability

The data that support the findings of this study are available from the Peripheral Neuropathy Research Registry (PNRR). Restrictions apply to the availability of these data, which were used under license for this study. Data are available from the authors at the URL with the permission of the PNRR and the Foundation for Peripheral Neuropathy.
